# Are We Facing a Radical Change in the Migration Behavior of Medical Graduates from Less Developed Countries? Demographic Profile vs. Social Push Factors

**DOI:** 10.3390/ijerph20064894

**Published:** 2023-03-10

**Authors:** Valentina Vasile, Elena Bunduchi, Daniel Stefan, Calin-Adrian Comes, Razvan Vasile, Anamari-Beatrice Stefan

**Affiliations:** 1 Institute of National Economy, Romanian Academy, 13 Calea 13 Septembrie, 050711 Bucharest, Romania; valentinavasile2009@gmail.com (V.V.); daniel.stefan@umfst.ro (D.S.); calin.comes@umfst.ro (C.-A.C.); 2 Faculty of Economics and Law, George Emil Palade University of Medicine, Pharmacy, Science, and Technology of Targu Mures, 38 Gheorghe Marinescu Street, 540139 Targu Mures, Romania; beatrice.stefan@umfst.ro; 3 “Costin C. Kirițescu” National Institute of Economic Research, Romanian Academy, 13 Calea 13 Septembrie, 050711 Bucharest, Romania; vasile.razvan89@gmail.com

**Keywords:** medical students, study migration, push factors, logistic regression, odds ratio

## Abstract

The phenomenon of migration among medical personnel from less developed countries is a large one, with negative effects on the origin country, but more worrying is graduates’ propensity to migrate during or immediately after university studies. The analysis of the labor market in the health sector from the last two decades shows us greater attractiveness of employment in (more) economically developed states compared to the demand from the health sector in graduates’ origin countries. This research’s purpose is to identify the determinants of the propensity to study and work abroad of medical students as a defined factor for better employment, and to identify the push factors in the origin country. As a result of the dichotomous nature of the dependent variables, logistic regression was applied. The independent variables (gender, residence, medical specialization, grades and perceived economic status) were used to identify the odds of the intention to migrate for studies. The results highlighted a higher propensity to migrate for studies among medical students, with opportunities offered by universities differing across countries and geographical areas. Moreover, students with a lower level of household income have openness to migrate, the tuition fees being managed through part-time/temporary employment during studies.

## 1. Introduction

The return of medical graduates to their country of origin, after studies abroad, is an exception, due to more favorable employment opportunities in the country where they complete their studies. In addition, postgraduate specializations abroad are most often associated with the decision to stay/enter the labor market of the host country. At the same time, the migration of doctors, and medical personnel in general, to medical units that offer better paid and more decent jobs, with appropriate support that allows for the development of a professional career, has become a reality in recent decades and higher attractive factor after the COVID-19 pandemic “experience”. The pandemic period proved the importance of the association of work factors for the quality of the medical service, namely: the need to ensure, quantitatively and qualitatively, human resources, the required equipment, the availability of treatments and other logistics and communication components specific to the medical system. Thus, it is worth mentioning that the reaction of governments of more developed countries was to simplify the process for inward migration and professional licensure to facilitate rapid recruitment of international personnel [1]. Less developed countries have also taken measures to attract graduates to employment in the health sector, including through a moratorium on the outward migration of health personnel during the COVID-19 pandemic [1,2] or by increasing the salaries of young people in the medical system, including for graduates’ employment (i.e., for Romania) [3,4,5,6,7,8].

So, it is obvious that, after the experience of the medical services offered during the pandemic, the employment model and health jobs’ requirements are changing significantly for most jobs and, radically, for health specialists—more flexible access, increasing the income gap compared with a similar position in the origin countries, higher opportunity for professional career and future mobility in OECD countries for better jobs, etc. [9,10,11]. Moreover, the need for adaptation, to anticipate skill needs in the health workforce, being user-oriented, contrasts with chronic lack of funding, insufficient inter-institutional coordination, poor stakeholder involvement, and asymmetry between skill intelligence and the desired policy purpose from less developed countries, creates pressure on external mobility for better training/specialization. This is also the reason why the motivation of mobility for work is being redefined, and the expectations of higher education graduates are radicalizing towards the employment model of the future workplace with adequate technological and logistical conditions, availability of information (with the support of databases managed with AI), and professional specializations in interdisciplinary approach addressed to the efficiency and quality of the medical service. Moreover, the facilities offered during the COVID-19 pandemic by the developed states of the EU, and not only, with a more efficient medical service, to hire young immigrant specialists, by making access barriers more flexible (recognition of health professional qualifications, administering additional exams, granting/extending residency permits, other specific documents, etc.) [12] has increased the interest of students to continue their studies abroad or employment after graduation on the host country’s labor market. From this perspective, the openness of the education through students’ mobility programs, i.e., Erasmus+ program creating an updated view, participation in study visits and exchange of experience, allows the identification of differences, respectively, showing the comparative advantages of education in more developed countries [13]; thus, it is natural for students in medical fields to continue their postgraduate training with residency training and complementary specializations—through master’s and professional/scientific doctorates—abroad. 

The health workforce shortages worldwide will continue, and the mobility from less developed countries with poor work conditions to better equipped and financed medical facilities will continue to be an important push factor. In today’s society, and especially in the future, technological innovation and digital transformation in health will continue. Under these conditions, the main barrier to excellent training/education, decent employment, and performance at work will remain the insufficient funding of the health sector in its various forms; therefore, graduates of medical specializations become much more responsible and selective in the development of their professional career, even from the residency stage, seeking, naturally, to capitalize on the best opportunities for training and medical practice. [14,15]. The practice of recent years has shown that this means mobility for employment and postgraduate training, starting from the residency period in more economically developed countries, with financial potential and/or adequate funding sources. From the perspective of the last two decades, we can appreciate that we are facing an exodus of specialists from healthcare units [16,17] with shortages of equipment and funding (from less developed countries), with already chronic imbalances in the employment of the medical sector [18], to medical units that offer the prospect of the future in excellence in health services, i.e., the smart association of technological transfer progress [19] with financial comfort through employment (so-called “decent employment”). Additionally, modern medicine has come to build a necessary balance between prevention and intervention as a form of efficiency for the quality of healthy life.

The analysis of the specialized literature regarding the determinants of the migration of medical personnel from low-resource countries shows us a complex of motivational factors based on Maslow’s hierarchy of human needs [20,21]: “financial safety needs” [22] and the desire for professional development through better training opportunities and research [23,24,25,26]. Moreover, the COVID-19 crisis strengthened the resolve to emigrate for a lot of medical students [27].

Even though most of the literature is based on future doctors’ intention to migrate for work [26], we tried to identify the students’ propensity to migrate for further studies and employment, especially for residency training, the importance of this stage in career development and consequently employment option being poorly documented. 

**Figure 1:**
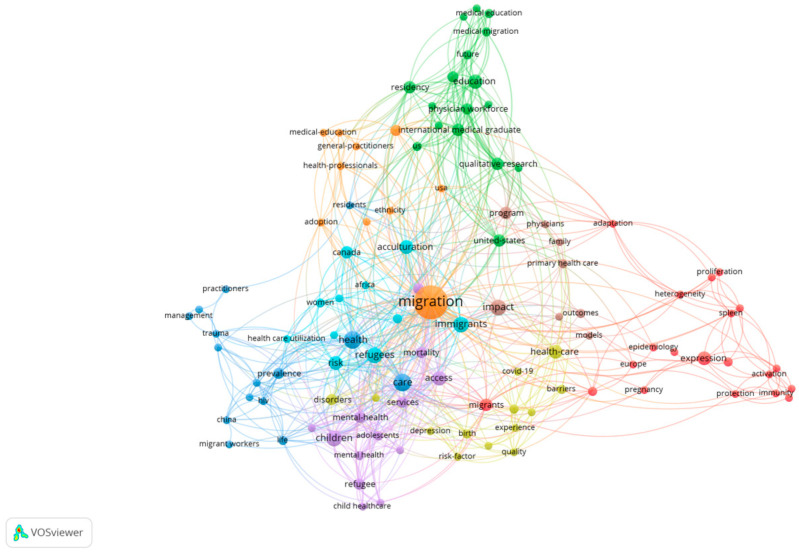
Research interest for graduates’ mobility with health specializations based on published works results in WoS, based on keywords Medicine or Dental Medicine or Healthcare or Pharmacy + migration + residency. Source: WoS database, accessed on 31 December 2022.

An analysis of the specialized literature by querying the Web of Sciences database (Figure 1) indicates a relatively small amount of published research on the issue of mobility for residency training stages of university medical graduates (just a few over 100 works) of which a half was only produced in the last years (2019–2022). This indicates that graduates migrate less only if they have completed their university studies, and the problems that arise are employment after the residency training stage.

The unanimous opinion of the specialists is that the motivation for mobility is provided by the clearly increasingly attractive opportunities for a professional career. If specialization internships target professional subfields and an education level/profile of interest to the host country, the professional career continues, most of the time through occupation in the host country [15,28,29,30,31]. In addition, for less developed countries, the motivation of substantially better remuneration than in the country of origin makes the investment in education for the health sector open to being capitalized on in a different market to the location of professional education/training.

The decision to analyze the changes in the intention to continue studies abroad derives from the diversity of incentives, the political support for the legislation regarding the increase of places financed for the continuation of studies, and salary increases for young residents in various less developed countries such Romania. In this sense, the question arises as to whether the migration trend for education and career is maintained, and if so, what are those determining factors, in the context in which the official statistics show that Romania, through the growing number of doctors it exports to the member states of the European Union or OECD, finance the medical systems there, by offering budding specialists with graduate studies from the resources of the public budget? 

The main issue is the background questions addressed by (a) the graduates, regarding professional career and high quality of medical services that are able to be performed, and (b) by the less developed countries’ policymakers on net benefits from a changing employment model for the graduates in order to increase motivation for returning after specialization abroad or to continue a residency training stage and after that have a job in their native country. The problem is more complex and difficult for less developed countries, especially after the “COVID-19 experience” [32,33], when all the countries of the world realized the value of financing excellent medical services [34], but also for incoming graduates, from the point of view of changing their perception towards expectations vs opportunities. In this work, we set out to identify the significant change, or not, in the perception of future graduates regarding their professional career and further training through professional specialization.

**Research hypothesis**

As a result of the importance of the migration phenomenon among medical personnel, especially following the COVID-19 pandemic, we consider it important to identify the determinants of the intention to migrate for studies, in an initial phase, of the medical graduates.

The traditional push factors [35] based on “the gap in the supply and demand in developed countries and a lack of job satisfaction in developing countries” [36], professional development opportunities [37], “aspirations and desires” [38] and higher incomes [39] also remains valid for the future.

In recent years, a series of studies have analyzed the determinants of labor migration [40,41], in general, and of medical personnel, in particular, both for work and for studies [42,43,44,45,46,47]. Extending the policy of attracting young people through improving working conditions for junior doctors [48], gender openness [49,50] or drivers defined by push–pull plus [51] will fuel the students/graduated propensity for mobility abroad. 

At the EU level, it is noted that in the field of health, the number of female students far exceeds the number of male students (almost three times at the EU27 level and more than twice in the case of Romania)—Table 1. 

For this simple statistical reason, it is expected that the number of those who tend to migrate will be higher among female students. Analyzing the dynamics of the presence of women in the health sector, we can see their higher proportion worldwide and the gradual reduction in the gender gap in recent decades, the reasons being multiple, from specialty choice (more “people-orientated” [53], “patient-centred care” [54,55]) to cost-efficiency (women are lower paid than men in similar positions, have higher percentage of part-time positions, etc.).

For the present research, we defined the following research hypothesis:

**H_1_.**  *Do female students tend to migrate more than males to continue their studies?*

According to the research literature [56], another determinant of the migration decision is place of residence. Deressa and Azazh [49] segregated the sample included in the analysis and found that students behave differently depending on their place of residence. So, the students from the rural environment are the ones who accept practice in the rural environment much more easily; respectively, their intention to migrate is diminished compared to those from the urban environment. At the same time, Hagopian et al. [57] found that urban students tend to migrate for studies or work in urban areas of developed states more than those from rural areas.

**H_2_.**  *Do medical students from urban tend to migrate more to continue their studies?*

In addition to the gender and place of residence of the graduates, another element of the demographic profile is the level of the graduates’ grades. The migration intention of young graduates is based on many social, professional, family and economic reasons, or the level of the educational system in the country of origin. Students who decide to continue their studies as a result of the low level of the educational system in their country of origin or because they want to evolve from a professional point of view are usually those with a high level of qualifications. From these considerations, the following research hypothesis emerges:

**H_3_.**  *Do students with higher grades tend to migrate more to continue their studies?*

If the economic motivation is significant in the decision to migrate for work, e.g., migrants having remittances as their goal in order to increase the standard of living of the family staying at home, migration for studies provides a new perspective of the economic factor. Students with a low level of income face difficulties in the migration process for studies. Most of the time, the expenses recorded in the countries of destination far exceed the level of those at home, and these expenses have a determining role in the migration decision [18]. For this reason, previously conducted studies [58,59] demonstrate the fact that wealthy students are more likely to migrate in order to continue their studies.

**H_4_.**  *Do students with above-average family incomes tend to migrate more to continue their studies?*

All of the identified determinants of the intention to migrate for studies influence the decision of medical graduates and transform Romania into a medical educational hub [60] for developed countries, where students complete their undergraduate studies and choose to continue their master’s or residency studies in another state or complete the entire cycle of studies in Romania (including residency studies) and choose to migrate for work. Thus, OECD data show Romania as one of the largest exporters of doctors and medical assistants in the EU [60,61].

**H_5_.**  *Do students with Medicine and Medical assistants’ specialization tend to migrate more to continue their studies?*

## 2. Materials and Methods

A cross-sectional study was conducted in medical universities in Romania, with the participation of final year students. This study consisted of applying a questionnaire distributed on the official information channels intended for medical students between September and December 2022. The questionnaire was built using the Google^®^ Forms option and was anonymous, without collecting personal information. Initially, the questionnaire was tested on 30 students, who were later not included in the final analysis.

The questionnaire was formulated taking into account research directions from the specialized literature, adapted to the needs identified in Romania. The first section of the questionnaire asks for demographic details, and in the case of a positive answer to the question on the intention to migrate for studies, respondents were directed to another section, asking for details on the reasons behind the decision and preferred destination states.

### 2.1. Materials 

The questionnaire was applied to students from the faculties of Medicine, Pharmacy and Dental Medicine, obtaining 456 responses in the initial phase. After eliminating the invalid questionnaires (those respondents who did not successfully pass the control questions), we had 392 validated responses.

Even though the responses to random online questionnaires are generally free of gender bias [62,63], we noticed a large gender gap between respondents, with much higher participation of women [64]. From the total number of respondents (Table 2), we found that the overwhelming majority is represented by women (77.8%), followed by only 20.7% men, and 1.5% of the respondents not wanting to mention their gender. This gender discrepancy of the respondents can be explained by the fact that in the field of health, the number of female students is at least twice as high compared to that of male students (Table 1). At the same time, it has been demonstrated that women are more receptive in responding to questionnaires, surveys or other qualitative data analysis tools [50,65]. Significant discrepancy was also registered in the case of the living environment: urban—85.5% and rural—14.5%. Most of the respondents were students specializing in medicine, followed by future dentists and pharmacists, and only 27 of future medical assistants answered. Among all the respondents, most considered the fact that they have good grades, comparing with their colleagues, and in terms of regarding the economic status, over 80% considered that their households have an average or above average income level.

In order to apply the logit model, we transformed all the results of the variables used into categorical data, according to Table 3.

### 2.2. Methods

A qualitative data analysis was further conducted due to the dichotomous nature of the dependent variable—intention to continue studies abroad. In order to test the presented research hypotheses, we applied a binary logit model, both as a result of the type of data analyzed, and as a result of the fact that the literature in this field predominantly uses this type of questionnaire analysis. Similar studies [29,31,66,67,68,69] that analyzed the determinants of the migration intention of medical university students or medical staff used logistic regression in their econometric analysis; thus, for the binary dependent variable, it is the most suitable method of econometric analysis [70].

The model can be generally written as:

(1) $$P[y_{i}=1\;|x_{1i},x_{2i},\;\ldots ,\;x_{ni}]=f\left(x_{1i},x_{2i},\;\ldots ,\;x_{ni}\right)$$

where:

– *y_i_* is the dependent categorical variable 1 if yes or 0 if no;
– *x_i_* are the independent categorical variables with different scales;
– *f*(·) is the logistic function.

The logit model can be estimated via maximum likelihood estimation (MLE) using the numerical method. 

Logistic regression must fit the data used in order to test the research hypotheses. Logistic regression quantifies the odds of increased intention to migrate for studies of medical students, according to the questionnaire results, using each of the independent variables (gender, residence, medical specialization, grades, and economic status), when others remain constant. Using the general formula for representing the logistic model, the independent variables obtained after applying the questionnaire and our research hypothesis, we built the following econometric models (Table 4): 

## 3. Results

Following the application of the logit regression for the five models (Table 5) we found that the medical students’ intention to migrate for studies was persistent and significant, not only from an economic perspective, but also from an econometric one.

Table 5 presents the odds coefficients for the five models, in which we analyzed the impact on the intention to migrate for studies considering gender, the environment in which the students have their residence, the specialization they follow, the personal perception of the level of grades they obtain and their economic status.

Regarding the gender variable, we found that both in the case of Model 4 and 5, female students tend to have a stronger tendency towards migration compared to respondents who do not want to mention their gender. At the same time, the intention to migrate for the continuation studies expressed by male students is not significant from a statistical perspective. Thus, in the case of Model 4, the odds of female students’ intention to migrate for studies is 8.95% higher compared to the reference respondents if the other variables remain constant. Additionally, in the case of Model 5, the odds of being female students increase the intention to migrate by 7.46% compared with no answer respondents if the other variables remain constant.

Moreover, we used another categorical variable—residence (Model 4 and 5). This independent variable, however, does not exert any influence on the decision to migrate for studies. Therefore, young people from the urban area do not have a stronger intention to migrate compared to those from the rural area.

The specialization followed within medical universities was used in Models 2, 3 and 5. In all cases, the results are similar; when all other variables included in the econometric model are constant, future medical students have the highest odds of migrating to other destination countries for studies compared to those from pharmacy (Model 2: odds ratio 2.39734; Model 3: 2.64368; Model 5: 2.49522) at a Pr(>|z|) < 0.05. Graduates of the dental medicine specialization do not have higher odds compared to those from pharmacy. Future medical assistants, similar to medical students, tend to continue their studies in another country (at a Pr(>|z|) < 0.1) if the rest of the independent variables remain constant.

Grade position, on the other hand, has no statistical influence on migration propensity.

The economic status perceived by each individual student influences the decision to migrate. Students with a good or average level of personal or household income (ref: very good) do not have a high migration propensity, but with the decrease by one unit from neutral to poor, the odds of migration for studies increases by approximately 30–40%, depending on the analyzed model, if the other independent variables remain constant.

## 4. Discussion

Our results show that the intention to migrate for studies among students from universities with a medical profile still persists, even if significant changes related to remuneration and opening of employment opportunities in public health were made. Given the deep employment crisis in the sector, with the higher asymmetric imbalance structure situation of the medical system in Romania, the propensity to migrate for studies causes concerns. The increased shortage of medical personnel in Romania, since before the COVID-19 pandemic, has led the authorities to implement some public policy measures including the number of residency training positions in healthcare units and higher salaries offered not only to specialist staff, but also to residents. All these measures are intended to retain the young university graduates with specialization in health on the labor market in Romania. However, it seems that the measures have not had the expected effect; as a result, both the shortage of doctors and the intentions to migrate for studies are still present. 

The shortage of doctors continues to exist not only because of the migration of specialized medical personnel, but also of graduates who want to continue their master’s studies and/or residency in another country of destination. If residency studies are not mandatory for pharmacists when they want to practice, having the possibility of employment in pharmacy chains (to occupy a job as a pharmacist in a hospital alone, pharmacists need residency studies), for the other categories of medical staff, such as dentists or doctors, things are different. For dentists (dentistry graduates can work without master’s studies or residency, but their activity is limited to a few simple medical operations, and if they want to provide orthodontic or dental surgery services, they are obliged to follow residency studies) and especially for doctors, residency studies are mandatory in order to obtain the specialization that allows them to practice. A doctor needs residency studies to become a specialist, and without them he has no right, according to the national legislation [71], to practice in any medical unit.

Analyzing the results obtained following the application of logit regression, we found that those who have the greatest tendency to migrate for studies are female students. Although the results obtained are in contradiction with some studies in the field, which present men as initiators of the migration process [72,73], in Romania, the situation is different, so H_1_ is accepted. The increased openness of women regarding the intention to migrate results from at least two reasons. The first is the demographic situation of the medical staff. In the period 2000–2021 (Table A1), the share of women is over 70% in the case of doctors and over 90% in the case of nurses. At the same time, in the case of the respondents, over 77% of them are women. The second reason is the divergent evolution of the graduates’ gender profile vs discrimination in employment. In Romania, female university graduates exceed the male number by over ten percentage points [74]. It is also worth mentioning that Romania is a country of paradox in the health sector. It registers the fewest graduates of higher education in the EU, compared to the number of inhabitants, but it has the highest number of medical graduates per 100,000 inhabitants in the world [75], namely 36 graduates in 2011, compared to only 6.5 in the USA and 9.3 in the UK. Moreover, in the last decade, fewer and fewer of them want to stay on the labor market in Romania, the reasons being mixed, from the significant salary differential to reasons related to the prospect of career advancement, the asymmetry of the employment offer, etc.

Odds of study migration do not change if we include residence in the analysis. Although there are studies according to which the tendency to migrate is much more frequent in the case of people from urban areas [76], in Romania, young people from rural areas who choose to stay in the country, after completing their studies, do not go back, but choose to practice in city hospitals, the gaps between the two areas being enormous (Table A2). Consequently, H_2_ is rejected, as this independent variable has no justifying role on the decision to migrate for studies.

Regarding the impact of the specialization pursued by students on the intention to migrate for studies, discrepancies were found. Thus, future doctors and medical assistants are more prone to migration compared to those in dentistry and pharmacy (H_3_ is accepted). The less developed a country is, the greater the number of pharmacies operating in the market and of the pharmacists per 100,000 inhabitants at the level of European countries (Table A3). According to a study carried out by ABDA (Federal Union of German Associations of Pharmacists) [77], Romania ranks 9th among the EU states, with over 40 pharmacies per 100,000 inhabitants. Thus, Romania ranks above the EU average (32 pharmacies per 100,000 inhabitants) or above developed EU countries, such as Denmark, Sweden, The Netherlands, Finland, Austria and others. This phenomenon is primarily due to the poor state of health of the population as a result of low investments in the medical system, as well as self-medication practices. People from less developed countries, especially those with a low level of income, with many children and without a stable job [78] and poor access to healthcare [79], tend to use the services of a pharmacist [80], which are free, rather than to go to a medical consultation, which should be paid for, if the person is not insured. In this situation, large pharmaceutical chains are increasingly being developed, and the graduates of the faculties of pharmacy do not lack jobs or low salaries, as happens in other cases. Their intention to migrate for studies, with the possibility of retention in the labor market of the destination country, is not justified, especially if the pharmaceutical sector is not as attractive in developed countries.

H_4_ is rejected, since grades have no influence on students’ propensity to migrate for studies.

Regarding the students’ economic level, we found that poorer Romanian students tend to migrate for studies, especially if they manage to obtain scholarships, even if the literature states otherwise [81], so H_5_ is rejected. The tendency to migrate among those with a lower level of income can be justified by the desire to rid themselves of poverty and increase the standard of living of family members through remittances.

The migration propensity of young graduates has a background behind a well-developed network of Romanian doctors, Romania being one of the largest exporters of medical labor for OECD countries OECD [10,11,82]. Thus, young graduates tend to make decisions regarding migration to continue their studies and even for employment in other countries of destination. 

**Figure 2:**
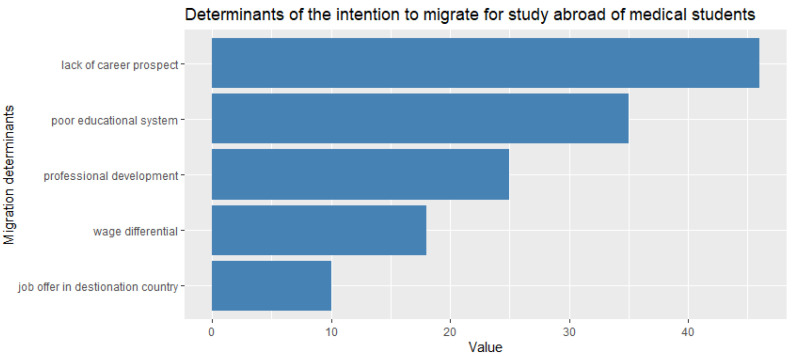
Determinants of the intention to migrate for study abroad of medical students. Source: questionnaire answers.

Analyzing the motivation behind the intention to migrate for studies, we found mainly push factors, which determine the students continuing their studies abroad (Figure 2). More than 45% of all respondents believe that, in Romania, they do not have a good career perspective in the medical system, even if the authorities have undertaken a series of economic measures aimed at retaining them. The lack of prospects for career development is followed by the gaps that students have identified in the Romanian education system, especially as a result of online courses due to the COVID-19 pandemic [37]. At the same time, some respondents consider that continuing studies abroad can contribute to professional development, similar to the migration motivation identified by Schumann [37], Gungor and Tansel [83] and Wilson [84]. Job offers in the destination states are the least important, which once again underlines the fact that it is the situation of the medical system that pushes students to migrate, and not necessarily the idea of migration itself.

**Figure 3:**
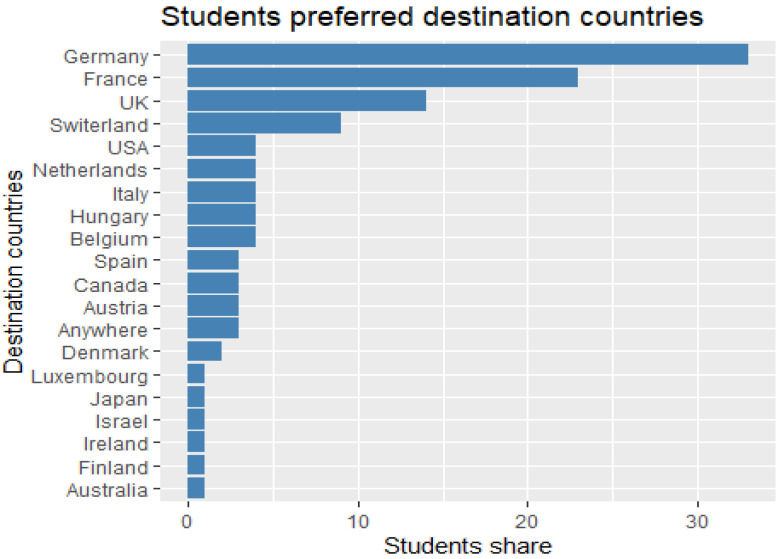
Romanian medical students preferred destination countries to study abroad. Source: questionnaire answers.

The countries preferred for further studies correspond to those chosen by specialist doctors in their decisions to migrate for work, according to OECD data [10,11]. Thus, among the preferred destination countries we found Germany (more than 30% of the respondents want to continue their studies there), France (23%) and the United Kingdom (14%), and 3% of the respondents would go anywhere just to leave Romania (Figure 3). 

In order to continue their studies in an EU member state, at the beginning, Romanian students only need the recognition of their previous studies. After three months, the country of destination can request registration of their residence, by demonstrating that they are enrolled in an educational institution, that they have sufficient financial resources and that they have comprehensive health insurance.

Those who wish to continue their studies in the USA or Canada, in addition to the recognition of studies and language certificates, must obtain a study visa, which can make it difficult for them to access these countries.

**Figure 4:**
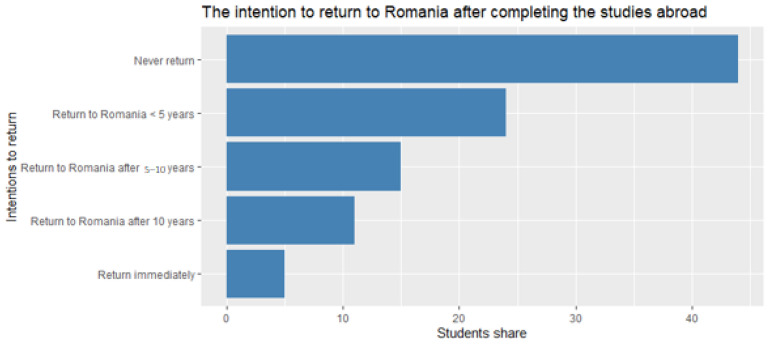
The intention to return to Romania after completing the studies abroad. Source: questionnaire answers.

More than that, and even more worrying, is the fact that almost half of those who want to continue their studies abroad do not intend to come back, so the type of migration changes from study migration to labor migration (Figure 4). On the other hand, only 5% of respondents want to return immediately after completing their studies. Thus, Romania ends up financing the exodus of medical personnel to developed states of Europe by covering the cost of undergraduate studies.

As a further research direction, we proposed to expand the questionnaire and increase the number of respondents. At the same time, we anticipated the expansion of the questionnaire by including questions related to the intention of labor migration, not only that of studies, as a result of the fact that there is a tendency to complete studies in the country, but with employment abroad.

## 5. Conclusions

The labor market in the health sector is strongly internationalized, despite the strict requirements for training and permits to practice. The flows of medical graduates who migrate from less developed states to developed ones, under the condition of the chronic shortage of medical personnel in the countries of origin, continue to increase. Public policies prove to be poorly effective in retaining graduates, the reasons being multiple, from remuneration, working conditions, career prospects and the stressful work environment in conditions of staff shortage, quantitative and structural. In these circumstances, although the pandemic period has highlighted the importance of employment in health and of the structure by specialization, associated with quality logistics services (equipment, drugs, etc.) and governments have taken measures to cover deficits, medical graduates remain selective in their decision of specialization and future employment, especially those from less developed countries, oriented towards identifying opportunities from external mobility.

The present research focused on the analysis of the graduates’ perception, who studied and had practical activity, as students in units of the health system in Romania, before and during the pandemic. The professional path—the residency period and employment on the labor market—were the objectives pursued in the development of the questionnaire, these aspects being, in the opinion of the authors, essential to their professional future.

The limits of this study are the small number of respondents, which may create problems of statistical representativeness. However, compared to final year medical students in Romania, at that level in 2022 (the questionnaire was applied in November–December), their number is significant to identify the social profile of the graduate looking for continuing education (residency studies) and future employment in the country of birth or abroad. Another limitation consists of the impossibility of verifying the fulfillment of the respondents’ intention to migrate for studies and the reasons behind the eventual change in the decision.

The obtained results indicate a faster adaptation of the future graduates to the changes generated by exogenous (increasing the quality and period of healthy life of the population, access to health and health inclusion for all) and endogenous challenges to the health system (reshaping the structural model due to pandemic) than that of decision-makers. The changes made by public authorities are so necessary to rebalance employment deficits of specialists and medical staff in general as a fundamental step in preparing the health sector for the future and increasing the quality of medical services.

A recommendation for the country of origin, without restricting the right to free movement for education and for a job, it is to rethink public policy measures to retain young doctors in the country of origin, both for studies and for work–communication, high-performance infrastructure and finally attractive salaries and working conditions.

## Figures and Tables

**Table 1:** Students enrolled in tertiary education in health programs by gender, persons.

	2013	2014	2015	2016	2017	2018	2019	2020
**European Union-27**								
Female	1,480,633	1,577,691	1,486,832	1,494,802	1,625,338	1,672,133	1,691,707	1,757,499
Male	588,388	634,037	617,661	630,191	674,804	662,336	669,498	686,250
**Romania**								
Female	52,294	49,925	45,871	46,876	49,070	50,477	50,895	52,432
Male	24,780	24,955	22,359	22,057	23,420	23,588	22,535	23,219

Source: Eurostat database [52].

**Table 2:** Demographic characteristics of respondents.

Demographic Characteristics	N (%)
Gender	
Female	305 (77.8)
Male	81 (20.7)
Area:	
Rural	57 (14.5)
Urban	335 (85.5)
Specialization	
Pharmacy	61 (15.5)
Medicine	237 (60.5)
Dental Medicine	67 (17.1)
Medical assistants	27 (6.9)
Grades position in class	
Very good	164 (41.8)
Good	214 (54.6)
Sufficient	14 (3.6)
Economic status	
Very good	28 (7.1)
Good	197 (50.3)
Neutral	133 (33.9)
Poor	28 (7.1)
Very poor	6 (1.6)

Source: questionnaire answers.

**Table 3:** Variable description.

Variables	Data Type	Variable Description	Possible Values	
study_abroad	Categorical unordered	Intention to continue studies abroad. Dependent answer variable	Yes	“0”
			No	“1”
gender	Categorical unordered	Gender	Did not answer	“0”
			Male	“1”
			Female	“2”
area	Categorical unordered	Area of living	Rural	“0”
			Urban	“1”
specialization	Categorical unordered	Medical specialization	Pharmacy	“0”
			Medicine	“1”
			Dental Medicine	“2”
			Medical assistants	“3”
grades	Categorical ordered	Grade position in class	Very good	“0”
			Good	“1”
			Sufficient	“2”
econ_status	Categorical ordered	Economic status	Very good	“0”
			Good	“1”
			Neutral	“2”
			Poor	“3”
			Very poor	“4”

**Table 4:** Logistic regression model description.

Models	Model Description
Model 1	$P[study\_abroad_{i}=1|grades_{i},\;econ\_status_{i}]=\frac{1}{1+e^{-\left(\beta_{0i}+\beta_{1}\cdot grades_{i}+\beta_{2}\cdot econ\_status_{i}\right)}}$
Model 2	$P[study\_abroad_{i}=1|grades_{i},\;specialization_{i}]=\frac{1}{1+e^{-\left(\beta_{0i}+\beta_{1}\cdot grades_{i}+\beta_{2}\cdot specialization_{i}\right)}}$
Model 3	$P[study\_abroad_{i}=1|grades_{i},\;specialization_{i},\;econ\_status_{i}]=\frac{1}{1+e^{-\left(\beta_{0i}+\beta_{1}\cdot grades_{i}+\beta_{2}\cdot specialization_{i}+\beta_{3}\cdot econ\_status_{i}\right)}}$
Model 4	$P[study\_abroad_{i}=1|gender_{i},\;area_{i},\;econ\_status_{i}]=\frac{1}{1+e^{-\left(\beta_{0i}+\beta_{1}\cdot gender_{i}+\beta_{2}\cdot area_{i}+\beta_{3}\cdot econ\_status_{i}\right)}}$
Model 5	$P[study\_abroad_{i}=1|grades_{i},\;area_{i},\;specialization_{i},\;gender_{i},\;econ\_status_{i}]=\frac{1}{1+e^{-\left(\beta_{0i}+\beta_{1}\cdot grades_{i}+\beta_{2}\cdot area_{i}+\beta_{3}\cdot specialization_{i}+\beta_{4}\cdot gender+\beta_{5}\cdot econ\_status_{i}\right)}}$

**Table 5:** Odds ratio logistic regression results (95% confidence interval).

Models	Model 1	Model 2	Model 3	Model 4	Model 5
Independent Variables					
Intercept	2.74254 *	1.94277 ***	2.72046 *	3.12840 *	2.66976
Gender (ref: did not answer)					
Male				0.78191	0.81719
Female				0.089589 *	0.074643 *
Area (ref: rural)					
Urban				1.03310	1.09060
Specialization (ref: pharmacy)					
Medicine		2.39734 *	2.64368 *		2.49522 *
Dental Medicine		0.84003	0.90372		0.8635
Medical assistants		2.65473 .	2.66974 .		2.90081 .
Grades (ref: very good)					
Good	1.24600	1.23437	1.24003		1.25722
Sufficient	0.78845	0.75686	0.74806		0.72936
Econ_status (ref: very good)					
Good	0.93331		0.80163	0.966897	0.82023
Neutral	0.73386		0.62720	0.79707	0.67854
Poor	0.37731 .		0.30137 *	0.367875 .	0.29509 *
Very poor	1.71567		1.13300	1.75838	1.19108

Note: significance threshold: ***—0.001; **—0.01; *—0.05; .—0.1.

## Data Availability

Data can be provided upon reasonable request.

## Appendix A

**Table A1:** Medical personnel by category and gender in Romania in 2000–2021, persons.

		2000	2005	2010	2015	2016	2017	2018	2019	2020	2021
**Doctors**	Total	45,786	47,388	52,204	56,110	57,304	58,583	60,585	63,303	65,740	68,760
	Female	30,530	32,543	36,151	39,052	39,892	40,992	42,606	44,566	46,334	48,527
	(%)	(66.68)	(68.67)	(69.25)	(69.59)	(69.61)	(69.97)	(70.32)	(70.40)	(70.48)	(70.57)
**Dentists**	Total	8307	10,249	12,990	15,556	16,442	15,653	16,457	17,003	18,491	19,982
	Female	5243	6624	8381	10,345	11,096	10,509	11,000	11,136	12,234	13,227
	(%)	(63.12)	(64.63)	(64.52)	(66.50)	(67.48)	(67.14)	(66.84)	(65.49)	(66.16)	(66.19)
**Pharmacists**	Total	7189	9283	13,624	17,135	17,180	17,833	17,620	18,093	19,470	21,470
	Female	6516	8505	12,410	15,349	15,392	15,992	15,822	16,160	17,387	19,290
	(%)	(90.64)	(91.62)	(91.09)	(89.58)	(89.59)	(89.68)	(89.80)	(89.32)	(89.30)	(89.85)
**Medical assistants**	Total	:	:	:	12,902	13,780	14,613	15,345	16,170	16,784	17,957
	Female	:	:	:	11,933	12,598	13,500	14,194	14,933	15,418	16,411
	(%)				(92.49)	(91.42)	(92.38)	(92.50)	(92.35)	(91.86)	(91.39)

Source: National Institute of Statistics data (2022) [85].

**Table A2:** The structure of the Romanian main categories of health personnel by place of residence, in 2020 and 2021.

Health Personnel Category	Medical Personnel Total		The Environment of Residence			
			Urban		Rural	
	2020	2021	2020	2021	2020	2021
Doctors (people)	65,740	68,760	60,154	63,300	5586	5460
- Inhabitants per 1 doctor	293	279	173	163	1592	1631
of which: family doctors (persons)	12,424	12,430	8098	8297	4326	4133
- Inhabitants per 1 family doctor	1551	1545	1282	1241	2055	2155
Dentists (persons)	18,491	19,982	16,330	17,790	2161	2192
- Inhabitants per 1 dentist	1042	961	636	579	4114	4063
Pharmacists (individuals)	19,470	21,470	16,047	17,898	3423	3572
- Inhabitants per 1 pharmacist	990	894	647	575	2597	2493
Auxiliary health personnel (persons)	73,931	76,060	65,466	67,592	8465	8468

Source: National Institute of Statistics data (2022) [86].

**Table A3:** Practicing pharmacists in European countries per 100,000 inhabitants, in 2012–2021.

Countries	2012	2013	2014	2015	2016	2017	2018	2019	2020	2021
Belgium	117.29	119	120.51	121.01	122.46	123.81	124.97	126.68	128.57	:
Bulgaria	:	:	:	:	:	:	:	84.45	87.91	88.21
Czechia	60.2	60.71	63.88	66.04	67.88	68.98	69.12	72.02	70.99	:
Denmark	49.86	50.74	51.01	51.57	52.01	53.13	54.02	44.32	:	:
Germany	62.74	63.77	64.22	64.35	63.67	64.7	65.73	66.69	66.95	:
Estonia	64.04	65.86	67.13	72.07	73.19	72.57	72.24	71.67	72.81	:
Ireland	:	:	:	:	:	:	:	107.39	:	:
Greece	:	:	:	:	:	:	:	:	:	:
Spain	105.19	111.75	117.4	119.28	120.83	115.61	118.88	123.33	131.61	:
France	106.1	105.78	105.75	105.55	104.69	103.53	102.81	102.72	101.84	:
Croatia	70.37	70.09	71.39	72.29	73.39	74.39	76.24	76.37	78.6	:
Italy	:	113.68	115.61	115.16	115.58	117.18	119.62	125.57	124.19	124.19
Cyprus	22.57	21.7	22.05	88.48	89.37	91.33	91.72	94.56	:	:
Latvia	68.92	80.09	77.64	79.59	83.85	95.25	85.62	84.28	86.51	87.21
Lithuania	:	:	:	:	:	99.1	102.8	102.89	102.62	:
Luxembourg	71.57	70.3	68.67	69.7	69.59	69.76	:	:	:	:
Hungary	57.29	76.05	76.97	71.51	74.92	77.21	80.22	83.13	78.41	:
Malta	108.09	110.1	113.45	125.38	132.86	129.27	128.76	129.35	135.83	:
Netherlands	20.55	21.22	21.55	21.25	21.44	20.84	21	21.46	21.64	:
Austria	69.07	69.79	70.19	70.63	70.9	71	71.82	72.96	73.08	:
Poland	70.52	72.15	73	74.03	77.08	77.24	:	:	:	:
Portugal	73.65	77.15	80.56	84.1	85.11	90.68	90.95	93.19	95.32	:
Romania	76.57	81.22	85.51	86.11	86.81	90.66	90.07	92.97	100.64	:
Slovenia	56.34	57.72	60.33	62.76	65.66	68.82	70.69	72.59	74.06	:
Slovakia	:	:	:	:	:	:	:	:	:	:
Finland	103.21	103.07	101.91	100.32	101.38	102.72	103.2	:	:	:
Sweden	77.03	75.87	76.25	76.92	77.15	76.96	78.93	79.38	:	:
Iceland	46.46	47.26	46.73	48.37	49.49	49.8	51.6	53.8	56.76	:
Norway	67.09	69.02	72.22	74.55	78.57	80.56	83.13	85.85	88.26	91.61
Switzerland	66.58	64.57	64.63	64.46	70.02	69.68	69.13	67.27	:	:
United Kingdom	78.07	80.02	82.44	83.51	86.18	88.3	89.83	:	:	:

Source: Eurostat data [87].
